# The Development and Application of a Base Editor in Biomedicine

**DOI:** 10.1155/2020/2907623

**Published:** 2020-08-14

**Authors:** Fang Wang, Yuqiang Zeng, Yi Wang, Yuyu Niu

**Affiliations:** ^1^Yunnan Key Laboratory of Primate Biomedical Research, Institute of Primate Translational Medicine, Kunming University of Science and Technology, Kunming, Yunnan 650500, China; ^2^Faculty of Life Science and Technology, Kunming University of Science and Technology, Kunming, Yunnan 650500, China

## Abstract

Using a base editor to generate monogenic disease models and correct pathogenic point mutations is a breakthrough technology for exploration and treatment of human diseases. As a burgeoning approach for genomic modification, the fused CRISPR/Cas9 with various deaminase separately has significantly increased the efficiency of producing a precise point mutation with minimal insertions or deletions (indels). Along with the flexibility and efficiency, a base editor has been widely used in many fields. This review discusses the recent development of a base editor, including evolution and advance, and highlights the applications and challenges in the field of gene therapy. Depending on rapid improvement and optimization of gene editing technology, the prospect of base editor is immeasurable.

## 1. Introduction

Benefit from the progress of gene therapy, we are entering an era in which genome editing tools could be used to manipulate gene sequences flexibly and precisely. Gene editing greatly drives the innovation of the treatment from symptoms to genetic basis of human genetic diseases. The first explosive event about gene editing came from Scherer and Davis in 1979, who develop a method that could be used to introduce foreign sequences into the chromosomes of yeast [1]. Then, researchers continuously finished precise gene targeting by homologous recombination in *Drosophila* [2], mouse [3], and human [4]. Afterwards, some engineered DNA-cleaving enzymes were discovered, including zinc-finger nucleases (ZFNs) and transcription activator-like effector nucleases (TALENs). Although both of them demonstrated the potentiality of therapeutic genome editing, they required a lot of time and labor. Subsequently, the development of clustered regularly interspaced short palindromic repeats-associated protein 9 (CRISPR/Cas9) offers a simpler technology which has been adopted widely, owing to its easier DNA-binding and modifying capabilities [5, 6]. CRISPR/Cas9 protein-RNA complexes were recruited to target DNA sequence via base pairing with a specified single guide RNA (sgRNA) and natively create a double strand breaks (DSBs), triggering cellular DNA repair by nonhomologous end joining (NHEJ) or homology-directed repair (HDR) to achieve genome editing eventually [7, 8]. Based on this property of CRISPR/Cas9, scientists have developed a variety of derivatives according to different gene editing requirements. For example, transcriptional repressors or activators were fused into catalytically inactivated Cas9 (dCas9) to achieve gene repression or activation [9–13]. In addition, in order to overcome the defect that conventional CRISPR/Cas9 induced abundant and unpredictable insertions or deletions (indels) and exhibited low efficiency in correcting point mutations, researchers developed a base editor—a new elegant Cas9 derivative which could efficiently generate precise point mutations with minimal indels. In this review, we will elaborate the development and application of a base editor in gene therapy.

### 1.1. The Evolution of Base Editor

#### 1.1.1. The Birth of Base Editor

Before 2016, researchers delivered CRIPSR/Cas9 with a donor DNA template to achieve gene correction. At present, the point mutations using HDR still remain inefficient (typically 0.1-5%), especially in unmodified or nondividing cell. The random indels around the cleavage sites are generally more abundant than gene replacement giving that the DSBs are preferentially repaired by nonhomologous end joining (NHEJ) in cells [6, 14].

**Figure 1:**
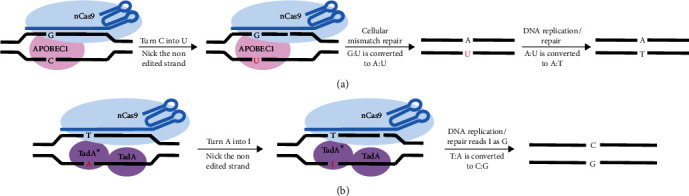
Schematic of CBEs and ABEs work in target DNA. (a) The working mechanism of BE3. Cytidine deamination by APOBEC1 enzyme that is tethered to the nCas9 converts the single-strand target C to U. Then, the BE3 nicks the nonedited strand containing the G, triggering DNA repair to induce G:U convert A:U. Eventually, A:U is converted to A:T during DNA replication or repair. (b) The working mechanism of ABEs. ABEs is composed of the fusion of TadA (wild type) and TadA∗ (TadA variant after protein evolution) and nCas9. The deoxyadenosine deaminase catalyses conversion of A to I, following DNA repair or replication by nicking in the nonedited strand. Eventually, the original T:A is replaced with C:G in the target site.

In 2016, Komor et al. added a new tool to genome editing toolbox, “cytidine base editor (CBE)”, which was a breakthrough in genome editing field. CBE can induce direct conversion of C to T or G to A at a programmable target locus without inducing DSBs and providing donor DNA templates. Importantly, CBE could significantly increase the efficiency of gene correction compared with HDR without introducing an excess of random indels. At the beginning, Komor and colleagues engineered a fusion protein-BE1, which mediate the direct conversion of cytidine (C) to uridine (U) within a five-nucleotide window specified by the sgRNA. The BE2 consists of three components, including a catalytically inactivated Cas9 (dCas9) derived from *Streptococcus pyogenes* Cas9 (SpCas9), a cytidine deaminase-APOBEC1, and an inhibitor of base excision repair-uracil glycosylase inhibitor (UGI). However, the result may be a reversal of G:U back to G:C, because the G:U mismatch can be corrected by unracil DNA glycosylase (UDG) which initiate the base excision repair (BER). They engineered BE3 by replacing the dCas9 with a catalytically impaired Cas9 (nCas9). BE3 nicks the nonedited DNA strand firstly, then converts G:U to A:U by activating cellular mismatch repair and finally converts A:U to A:T permanently during DNA replication and repair [15] (Figure 1(a)). Subsequently, they modified the base editor by installing mutations into third-generation base editor (HF-BE3) [16] or fusing base editor with a bacteriophage Mu protein which can bind DSBs and greatly reduce indels formation [17, 18]. Then, in 2017, David Liu and coworkers demonstrated adenine base editor (ABE), which enables the direct A to G or T to C translation. The ABE contains a modified deoxyadenosine deaminase (TadA∗), a wild type TadA, and an nCas9. Firstly, ABEs bind the target DNA guided by sgRNA. Then, the deoxyadenosine deaminase domain catalyzes the conversion of adenine (A) to inosine (I). Within the constraints of a polymerase active site, the inosine would be read or replicated as G. Eventually, the T: A base pair can convert to C: G base pair permanently [19] (Figure 1(b)).

To further increase the editing efficiency of the base editor (CBE and ABE), Koblan et al. optimized the nuclear localization signals (NLS) and codon usage, as well as reconstructed the ancestral deaminase component [20]. Up to now, the newest versions of base editors are BE4max, AncBE4max, and ABEmax.

With the emergence of CBEs, other two teams reported new base editor-targeted AID-mediated mutagenesis (TAM). They fused activation-induced cytidine deaminase (AID) or AID ortholog PmCDA1 with nuclease-inactive CRISPR/Cas9 for efficient genetic modifications, which enabled to perform highly efficient site-directed mutagenesis and high-throughput screening of functional variants [21–23].

#### 1.1.2. The Advance of Base Editor

Although base editor can help us to convert bases easily, there are still some problems needed to be addressed. The requirement of editing window and protospacer adjacent motif (PAM) greatly limits the scope of base editor.

When there are multiple editable Cs or As within or nearby the “editing window” (positions 4-8 for CBE or 4-7 for ABE, counting the PAM as positions 21-23), base editor could induce the conversion of bases edit in addition to the target base. To solve this problem, researchers further optimized the cytidine deaminase domains via inducing specific mutations, which eventually narrowed the width of the editing window from ~5 nucleotides to as little as 1-2 nucleotides [24]. For instance, YE1-BE3, YE2-BE3, EE-BE3, and YEE-BE3 are modified versions of BE3 with narrower active windows, but still show stable activity of base editing compared to regular BE3. Besides, the team of Tan obtained two high-precision base editors that BE3-PAPAPAP mainly edits within an activity window from −14 to −16, and base editors with CDA1 truncations mainly edit at position −18 [25, 26]. Conversely, in some cases, the editing windows need to be expanded to achieve targeted base editing. The groups of Jiang and coworkers developed base editor (BE-PLUS) with expanded C to U (T) programming scope [27]. Either narrower or broader strategy both enlarged the genome-targeting scope.

Except editing window, the PAM requirement also limits the number of editable sites. To broaden the targetable genome sequences of base editor, scientists have exploited numbers of Cas9 variants or homologue. Kim and coworkers, respectively, replaced the regular SpCas9 with four Cas9 variants to generate VQR-BE3 (NGAN), EQR-BE3 (NGAG), VRER-BE3 (NGCG), and SaKKH-BE3 (NNNRRT) [24]. Moreover, they used phage-assisted continuous evolution method to evolve a new SpCas9 variant (xCas9) with an expanded PAM including NG, GAA, and GAT [28]. Meanwhile, to break the G/C-rich protospacer-adjacent motif (PAM) restriction, the team of Jia Chen developed a CRISPR-Cas12a-based BE. They fused the rat cytosine deaminase APOBEC1 with a catalytically inactive version of *Lachnospiraceae bacterium* Cas12a (also named Cpf1) to achieve C to T conversion in human cells with a T-rich PAM [29]. Moreover, two team demonstrated new CBE variant (eA3A-BE3) which replaced the regular cytidine deaminases—rAPOBEC1 with human APOBEC3A—that have narrower editing windows that can reduce bystander mutations and mediate efficient C to T conversion in regions with high methylation levels [30, 31]. Recently, Richter and coworkers developed a new ABE variant—ABE8e—which activity has been increased 590-fold than ABE7.10's. ABE8e offers substantially improved editing efficiencies when paired with a variety of Cas9 or Cas12 homologs [32]. Up to now, there are several base editors' variants have been developed. These variants not only expand the editable range but also improve the editing efficiency of target sites (Table 1). All the variants hold great potential for both basic research and clinical application in biomedicine.

**Figure 2:**
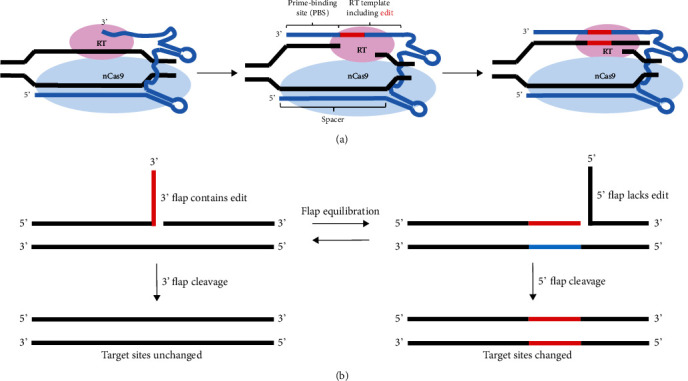
Schematic of prime editor works in target DNA. (a) The PE-pegRNA complex binds the target DNA and nicks the PAM-containing strand. The target strand's 3′ end is hybridized with the primer-binding site, then primers reverse transcription of new DNA containing the desire edit using the RT template of the pegRNA. (b) Flap equilibration in prime editing. Left panel represents the failure of hybridization. The 3′ DNA flap containing edited information is excised, resulting in target sites unchanged. Right panel shows the hybridization to DNA unmodified strand, and DNA repair process introduces mutation to the second DNA strand.

Moreover, in order to treat genetic disorders which were caused by multiletter mutations, such as Tay-Sachs disease caused by an insertion of four DNA letters into the *HEXA* gene [33], Anzalone et al. developed the prime editing (PE), a “search-and-replace” genome editing technology that mediates targeted insertions, deletions, and all 12 possible base-to-base conversions without requiring DSBs or donor DNA templates [34]. The new editor, PE, consists of an nCas9, a reverse transcriptase (RT), and a prime editing guide RNA (pegRNA). It can directly copy genetic information from the pegRNA into the target genomic locus (Figure 2). Because nicking the nonedited strand favors repair of that strand, resulting in preferential generation of desire replacement in cells, they developed PE3 which uses the Cas9 H840A nickase to nick the nonedited strand to further increase editing efficiency [34]. However, when a single target nucleotide is present within the base editing window, or when bystander edits are acceptable, primer editor is little efficient and generate more indels than current base editor.

Except in DNA level, base editing in RNA can also provide powerful capabilities for life sciences. To date, researchers had developed several base editors which can deaminate A to I, depending on the characteristic of ADAR family. The ADAR can mediate endogenous conversion of adenosine to inosine via hydrolytic deamination. The inosine is functionally equivalent to guanosine in the process of translation and splicing of the cell's protein building [35, 36]. In the early days, researchers developed an RNA editor that linked the catalytic domain of an ADAR enzyme to a guiding antisense RNA oligonucleotide [37–42]. Therefore, the ADAR deaminase domain (ADAR_DD_) can be recruited into the target RNA, which relies on the Watson-Crick base pairing between the antisense RNA and the target transcript.

**Figure 3:**
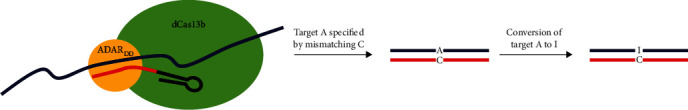
Engineering dCas13b-ADAR fusions for RNA editing. Schematic of RNA editing by dCas13b-ADAR_DD_ fusion protein which naturally deaminates adenosines to inosines in the target RNA. The crRNA specifies the target site by hybridization firstly, then creates a double strand RNA (dsRNA) structure and recruits the dCas13b-ADAR_DD_ fusion protein to deaminize. Besides, a mismatched cytidine in the crRNA is opposite the target adenosine which can enhance the conversion of target A to I.

In 2017, Shmakov's team developed a precise and flexible technology, Programmable adenosine to inosine Replacement (REPAIR), in RNA level by using the type VI CRISPR-associated RNA-guided RNase Cas13 [43–45]. RERAIR includes a catalytically dead RNA-guided Cas13b enzyme (dPspCas13b), an ADAR, and a sgRNA. CrRNA is targeted to the specific site by hybridization to create a dsRNA structure and recruit dCas13b-ADAR_DD_. And a mismatched cytidine in the crRNA opposite the target adenosine could enhance the editing reaction [46] (Figure 3). Except REPAIR, RNA base editing tools are also included, (RNA Editing for Specific C to U Exchange) RESCUE [47] and (Leveraging Endogenous ADAR for Programmable Editing of RNA) LEAPER [48], and they all mediated by ADAR enzymes in mammalian cells. The RNA editing allows a temporary correction of a disease-causing mutation without permanent alteration to the genome and could be a potentially safer option when it comes to gene-fixing therapeutics. At the same time, RNA editing can also help us interrogate genes and noncoding RNA as well as control cellular processes at the transcript level.

### 1.2. The Application of Base Editor in Biomedicine

#### 1.2.1. Disease Modeling

Base editor can induce specific base changes without DSBs and donor templates, which make it a convenient, high-efficiency approach for engineering nucleotide substitutions at target sites. There have been numerous reports showing that single-base editing systems can be successfully applied to bacteria [59, 60], plants [49, 61–64], zebrafish [65, 66], mammals, and even human [50, 67–74]. These studies all demonstrated the power of base editor in drug target research, crop improvement, animal disease modeling, gene function screeming, disease treatment, and so on. This review will focus on the modeling and treatment of different disease to describe the prospect of base editor in biomedicine.

In 2017, Kim's team firstly showed that CBE could be an efficient method to generate mice models with targeted point mutation [68]. Secondly, they proved that ABEs can also be used to generate disease mice models that obtain *Tyr* mutant with albino phenotype [70]. Furthermore, Li's team demonstrated that CBE or ABE system can be applied to generate rabbit models with the high mutation efficiency of 44-100%. They used CBEs to induce C to T conversion to generate a premature stop codon in *Mstn* and *Tyr* gene, respectively, and obtained two models that were double-muscled and albinism diseases. They also used CBEs to induced C to T conversion in *LMNA* gene leading in creating a cryptic splice donor site that produces a mutant lamin A protein, “progerin,” obtaining the third model which was Hutchinson-Gilford progeria syndrome (HGPS). Then, they used ABE7.10 to generate A to G conversion in *Dmd* (T279A) and obtained X-linked dilated cardiomyopathy (XLCM) model [69]. They got four kinds of disease models collectively, and the mutant rabbits showed the typical phenotypes observed in patients. Intriguingly, Liu and coworkers created mouse model harboring multiple mutations by using a combination of ABE and SaBE3. The mouse models recapitulated respective clinical defects and proved the specificity of ABE [75]. The study of Xie's group also showed that CBEs could induce C to T conversions at multiple sites in pig embryos simultaneously, and the mutation efficiency approximated 40~50% [67]. These studies mentioned all prove that base editor can be applied to generate mammal's models, which could mimic the mutations associated with human disease and could be used to guide the treatment of disease to some extent.

#### 1.2.2. Disease Treatment

To explore the feasibility and safety of base editor in gene therapy, researchers first studied in mammalian genetic disease models. Ryu's team demonstrated that delivering ABEs via transsplicing adeno-associated viral vectors to muscle cells in a mouse model of Duchenne muscular dystrophy enables the correction of the pathogenic mutation in the *Dmd* gene [70]. Two studies in nature medicine demonstrated that the base editor could be used to treat genetic disease in mice model of human autosomal recessive liver disease phenylketonuria or hereditary tyrosinemia type 1 [76, 77]. Recently, Thomas Gaj and coworkers established an intein-mediated transsplicing system that could deliver CBEs in vivo. They injected dual AAV particles encoding a split-intein CBE, introducing a nonsense-conding substitution into a mutant SOD1^G93A^, and achieving significantly slowed progression of ALS disease in mouse model [78].

Further, verifying the safety of base editor in clinical gene therapy, researchers are now focusing on human embryos and cells. In 2017, Huang's team reported the efficient correction of *HBB* (28 A>G) mutation in human primary cells and human embryos by BE3 or BE3's variants with corresponding sgRNA [50]. The *HBB* gene (28 A>G) mutation caused a common genetic disease, *β*-thalassemia, which is a major problem of global health. Researchers found that the mutation in *HBB* gene will lead to the reduction of hemoglobin *β* chain (*β*-globin) and erythrocytes, finally inducing oxygen shortage, bone deformity, organ dysfunction, and even organ failure in many parts of the human body [79]. At present, although the *β*-thalassemia patients could get treatment with blood transfusion and iron chelation, they still got numerous complication such as arrhythmia and hypothyroidism. Even the only curative therapy, bone marrow transplantation, is also limited by the antigen compatibility of human leukocyte. So, the disease is extremely lethiferous in humans currently. Huang's studies proved that using base editor in anemia could not only cure the disease but also prevent the disease from being passed onto future generations. Geurts and coworkers applied SpCas9-ABE and xCas9-ABE on four cystic fibrosis (CF) organoid sample. Their studies showed that both genetic mutations and functional disorders were repaired in all four cases, indicating that 20% of 664 patients in CF intestinal organoid biobank can be repaired by ABE [80].

At present, there have many prominent cases of base editor used in gene therapy for genetic disease (Table 2). Li and partners have successfully applied base editor in a cancer treatment for primary glioblastomas (GBM). The 124C>T in *TERT* gene would increase telomerase promoter activity and lead to the overexpression of TERT and preservation of telomere, enabling tumor cells to proliferate and evade senescence eventually. And it had been confirmed that there are 83% of existing *TERT* (124C>T) mutation lesions in GBM [81]. They developed a base editor variant which is composed of an nCas9 of *Campylobacter jujuni* and an adenine base editor (CjABE). They utilized CjABE to correct the 124C>T *TERT* promoter mutation. The local injection of adeno-associated viruses expressing *TERT*-specific sgRNA and CjABE could reduce the *TERT* transcription and protein expression by blocking the binding of members of the E26 transcription factor family to the *TERT* promoter, eventually facilitating the senescence and proliferative arrest of cancer cells [54].

All the studies demonstrate that the base editor can correct pathogenic gene mutations and have great prospect in gene therapy.

### 1.3. The Challenges of Base Editor

As with conventional CRISPR/Cas9 technology, there are two major bottlenecks that are off-target and delivery methods when applying base editor in practical applications. Although base editor cannot produce as many deletions and complex genomic rearrangements as that CRISPR/Cas9 does [88, 89], there are some shortcoming. Due to the property of deaminases which can modify RNA and single-stranded DNA at sites other than the intended target, the base editor can alter the DNA. Last year, two papers in science both reported the high levels of genome-wide off-target effects by CBEs [90, 91]. Yang and coworkers developed the Genome-wide Off-target analysis by Two-cell embryos Injection (GOTI) to detect off-target mutations. They injected CRISPR/Cas9 or base editor (CBE or ABE) into two-cell stage and compared the WGS results of edited and nonedited blastomeres at E14.5. Their study showed that the off-target single-nucleotide variants (SNVs) were rare in embryos of either CRISPR/Cas9 or ABEs, and the frequency close to the natural mutation rate. Surprisingly, the number of SNVs in embryos edited by CBEs was over 20-fold higher than that in others. Jin's team demonstrated that CBEs but not ABE induced substantial genome-wide off-target mutations which were mostly the C to T conversion by comparing the WGS results from rice plants edited by CBEs (BE3 and HF-BE3) or the ABE, with unedited population as control [90]. Moreover, the study of McGrath and coworker also revealed there were lots of unintended point mutations in human stem cells edited by CBEs [92]. Significantly, the three teams all demonstrated that the absence of sgRNA did not change the levels of nonspecific off-target edited by CBEs. The teams of Yang and Gao both showed an enrichment of SNVs located in highly transcribed genes. So, the result indicated that the APOBEC1 or UGI elements maybe responsible for the substantial off-target, because, in the natural state, APOBEC1 can bind single-stranded DNA (ssDNA) [93], and UGI can increase the spontaneous mutation rate [94, 95]. The random encounters between the deaminase domain of base editor and transient ssDNA may induce random nondirected off-target base editing [96]. Hence, decreasing the ability of APOBEC1 binding to ssDNA or the high levels of UGI may be good choices to reduce SNVs [97]. Recently, the team of Doman focused on the deaminase domain of APOBEC1 and engineered YE1 variants to narrow the on-target base editing window by screening of deaminase mutant. The new variants retain the substrate-targeting scope of high-activity CBEs as well as maintain minimal numbers of Cas9-independent off-targets [98]. In addition, the increase sensitivity of Cas9_R63A/q768A variant to mismatches within the target DNA maybe another good way [99]. Surprisingly, Kim and cooperators showed that except converting adenine to guanine, ABEs can also convert cytosine to guanine or thymine in narrow editing window and in a confined TC∗N sequence context [100]. Moreover, two papers in nature verified that base editor could induce off-target in RNA. Grunewald's team found that both CBEs and ABEs can cause extensive transcriptome-wide RNA edits in human cells and that CBEs-induced RNA editing occurs in both protein-coding and non-protein-coding sequences [101]. Zhou and coworkers also demonstrated that BE3 and ABE7.10 produced thousands of off-target in RNA level [102]. All of the studies warm us to seriously consider the problem of off-target before clinical therapy. Until now, there are several approaches that could be used to predict off-target sites [103–105]. Nevertheless, the predictions are usually far different from the WGS—a cumbersome and expensive approach. So, we need to develop reliable predictive software.

The other area that needs to be optimized is delivery strategy. Four general methods for delivery are electroporation, lipofection, viral vectors, and nanoparticles. Electroporation and lipofection are the primary methods used in vitro. Electroporation involves pulsing cells with high-voltage currents that create transient nanometre-size pores in the cell membrane to facilitate the delivery of base editor to cells. However, due to the particularity of operation, electroporation is limited to cell transfection in vitro. Lipofection reagent wraps plasmid vector DNA, forming DNA-lipid complex which could be absorbed via endocytosis of cell membrane, but the toxicity can cause massive cell death. Nanoparticle is another alternative way to deliver base editor via endocytosis and micropinocytosis. And nanoparticle is inexpensive and relatively easy to produce rather than the first two modes of transmission. However, this approach induces marked toxicity and show sensitivity in specific cell that limit the application. Adeno-associated virus (AAVs) is the most commonly used clinical delivery vehicle for gene therapy by the mechanism that viruses infect cells. The advantages are nongenomic integration and broad tissue targeting possibilities. Nevertheless, there are considerable challenges need to be addressed, which are uncontrollable immunogenicity, packaging capability, and high production cost of AAVs. Notably, the different forms of base editor also affect editing efficiency. Predictably, if the base editor stays in the cell too long, it will cause more off-target. Therefore, using the preassembled CRISPR/Cas9 RNPs with sgRNA can reduce possible off-target mutations due to the short half-life [16, 106, 107].

## 2. Conclusions

Gene editing is fascinating the medicine of the future and opening a window to actual personalized precision medicine. Depending on the character that CRISPR system could be anchored to target DNA or RNA sequences with relevant gRNA, base editor shows precise and highly predictable nucleotide substitution at target sites without DSBs and donor templates following little indels. The base editor guarantees the stability of the genome to some extent, when they are applied for gene editing. With the expansion of the application range of base editor, its accuracy and security need to be further ameliorated. As described in this review, two strategies could significantly reduce the off-target effect by reducing the intimacy between the base editor and nontarget site and optimizing the delivery method. There is no doubt that the base editor provides a powerful strategy for exploring the mechanisms and treating monogenetic disease, which have the potential to broadly impact the biomedicine.

## Figures and Tables

**Table 1:** Overview of different base editor variants.

First author	Publication years	Species	Category of base editor	Cas protein	Deaminase	Editing windows	PAM
Yuan Zong [49]	2017	Rice, wheat, and maize	PBE	nSpCas9	rAPOBEC1	3-9	NGG
Puping Liang [50]	2017	Human	YEE-BE3	nSpCas9	rAPOBEC1-YEE	5-6	NGG
Y Bill Kim [24]	2017	Human	SaBE3	nSaCas9	rAPOBEC1	3-12 for SaBE3	NNGRRT for SaBE3
			SaKKH-BE3	nSaCas9-KKH		3-15 for SaKKH-BE3	NNNRRT for SaKKH-BE3
			VQR-BE3	nSpCas9-VQR		4-11 for VQR-BE3 and	NGAN for VQR-BE3
			EQR-BE3	nSpCas9-VRER		EQR-BE3	NGAG for EQR-BE3
			RER-BE3			3-10 for VRER-BE3	NGCG for VRER-BE3
Pranam Chatterjee [51]	2018	Human	ScCas9-ABE7.10	ScCas9(n)	TadA-TadA∗	4-9	NNGN
Noah Jakimo [52]	2018	Human	Spy-mac nCas9-BE3	nSp-macCas9	rAPOBEC1	4-7	NAAN
Hiroshi Nishmasu [53]	2018	Human	Target-AID-NG	SpCas9(n)	PmCDA1	2-8	NG
Xiao Wang [31]	2018	Human	hA3A-BE3	SpCas9(n)	hAPOBEC3A	2-13	NGG
Xiaosa Li [29]	2018	Human	dCpf1-BE	dLbCpf1	rAPOBEC1	8-13 for dCpf1-BE and dCpf1-eBE	TTTV
			dCpf1-BE-YE		rAPOBEC1-YE	10-12 for dCpf1-BE-YE and dCpf1-eBE-YE	
			dCpf1-eBE				
			dCpf1-eBE-YE				
Johnny H. Hu [28]	2018	Human	xCas9-BE3	nxCas9	APOBEC1	4-8	NG, GAA and GAT
			xCas9(3.7)-ABE		TadA-TadA∗		
Xinjian Li [54]	2020	Human	CjABE	nCjCas9	TadA-TadA∗	9	GGGGACC
Shannon M. Miller [55]	2020	Human	CBE-NRRH	nSpCas9-NRRH	APOBEC1 for CBE	4-8 for CBE	NRRH
			CBE-NRCH	nSpCas9-NRCH	TadA-TadA∗ for ABE	4-7 for ABE	
			CBE-NRTH	nSpCas9-NRTH			
			ABE-NRRH				
			ABE-NRCH				
			ABE-NRTH				
Michelle F. Richter [32]	2020	Human	SpABE8e	nSpCas9	TadA-8e	4-8 for SpABE8e	NGG for SpABE8e
			SaABE8e	nSaCas9		3-14 for SaABE8e	NNGRRT for SaABE8e
			LbABE8e	dLbCas12a		8-14 for LbABE8e and enAsABE8e	TTTV for LbABE8e and
			EnAsABE8e	denAs-Cas12a			enAsABE8e
Xiang Lin [56]	2020	Human	SaCas9n-KKH-miniABEmax	nSaCas9-KKH	TadA∗	N.R.	NNNRRT
Ziying Hu [57]	2020	Human	SauriBE4max	nSauriCas9	APOBEC1 for CBE	4-13 for CBE	NNGG
			SauriABEmax		TadA-TadA∗ for ABE	6-14 for ABE	
Russell T. Walton [58]	2020	Human	SpG-BE4max	nSpCas9	rAPOBEC1 for CBE	3-9 for CBE	NGNN for SpG
			SpG-ABEmax		TadA-TadA∗ for ABE	5-7 for ABE	NRN for SpRY (R is A or G)
			SpRY-CBE				
			SpRY-ABE				

**Table 2:** Summary of application of base editor in gene therapy.

First author	Publication years	Species (tissues)	Category of base editor	Delivery	Disease	Gene	Mutation efficiency	Off-target
Alexis C. Komor [15]	2016	Mouse astrocytes	BE3	Plasmids	Alzheimer's disease	*APOE4*	58-75%	4.6–6.1% indels at the targeted locus
Alexis C. Komor [15]	2016	Human cell	BE3	Plasmids	Breast cancer	*P53*	3.3-7.6%	≤0.7% indel formation
Puping Liang [50]	2017	Human embryos	YEE-BE3	Injecting mRNA and gRNA	*β*-Thalassemia	*HBB*	22.9%	No off-target in top 10 predicted sites
Alexandra C. Chadwick [82]	2017	Adult mice	BE3	Adenoviral vectors	High blood cholesterol	*PCSK9*	Average 24%	≤1% indel formation
Seuk-Min Ryu [70]	2018	Adult mice	ABE	Adeno-associated virus	Duchenne muscular dystrophy	*DMD*	3.3 ± 0.9%	No off-target mutations at homologous sites with up to three mismatches
Luke W Koblan [20]	2018	Human fibroblasts	BE4, BE4max–P2A–GFP, AncBE4max–P2A–GFP	Plasmids	Glycosylation type 1f	*MPDU1*	32-77%	N.R.
Lukas Villiger [76]	2018	Adult mice	n*Sa*KKH-BE3	Adeno-associated virus	Autosomal recessive liver disease phenylketonuria	*PAH*	Average 25.1%	No C∙ G to T∙ A conversions or indel formations in ten potential off-target loci
			dLbRR-BE					
Alexandra C. Chadwick [83]	2018	Adult mice	BE3	Adenoviral vectors	Hyperlipidemic	*ANGPTL3*	35%	No off-target in top 10 predicted sites
Yanting Zeng [84]	2018	Human embryos	BE3	Injecting mRNA and gRNA	Marfan syndrome	*FBNA*	89%	No off-target and indels were detected in 32 potential off-target sites
Maria Paz Zafra [85]		Mouse intestinal organoids	FNLS-BE3	Plasmids	Colorectal cancers19	*Apc*	>97%	Less than 1% indels
Xinjian Li [54]	2020	Human U87 and U251 cells	CjABE	Adeno-associated virus	Glioblastoma	*TERT*	70-80%	N.R.
Xiang Lin [56]	2020	Human SMA motor neurons	SaCas9n-KKH-ABE	Injecting mRNA and gRNA	Spinal muscular atrophy	*SMN2*	5-40%	No detectable off-target effects in DNA level
Colin K.W.Lim [78]	2020	Adult mice	BE3	Adeno-associated virus	Amyotrophic lateral sclerosis	*SOD1*	N.R.	N.R.
Chun Qing Song [86]	2020	Adult mice	ABE6.3	Plasmids	Tyrosinemia	*FAH*	9.5 ± 4.0%	One off-target site
Jonathan M. Levy [87]	2020	Adult mice	BE3	Adeno-associated virus	Niemann-pick disease	*Npc1*	10-80%	One off-target site
